# On Modeling Missing Data in Structural Investigations Based on Tetrachoric Correlations With Free and Fixed Factor Loadings

**DOI:** 10.1177/00131644221143145

**Published:** 2022-12-20

**Authors:** Karl Schweizer, Andreas Gold, Dorothea Krampen

**Affiliations:** 1 Goethe University Frankfurt, Germany

**Keywords:** missing data, incomplete data, planned missing data design, structural investigation, tetrachoric correlation, confirmatory factor analysis, fixed factor loadings, free factor loadings

## Abstract

In modeling missing data, the missing data latent variable of the confirmatory factor model accounts for systematic variation associated with missing data so that replacement of what is missing is not required. This study aimed at extending the modeling missing data approach to tetrachoric correlations as input and at exploring the consequences of switching between models with free and fixed factor loadings. In a simulation study, confirmatory factor analysis (CFA) models with and without a missing data latent variable were used for investigating the structure of data with and without missing data. In addition, the numbers of columns of data sets with missing data and the amount of missing data were varied. The root mean square error of approximation (RMSEA) results revealed that an additional missing data latent variable recovered the degree-of-model fit characterizing complete data when tetrachoric correlations served as input while comparative fit index (CFI) results showed overestimation of this degree-of-model fit. Whereas the results for fixed factor loadings were in line with the assumptions of modeling missing data, the other results showed only partial agreement. Therefore, modeling missing data with fixed factor loadings is recommended.

Modeling missing data are a method of dealing with incomplete data sets in structural investigations. Incompleteness of data sets is a problem in empirical research with participants as suppliers of data. Following established conventions, we refer to what is missing as *missing data* but also use the short-cut *missings* where appropriate. There are different types of missing data: missing data that are completely at random (MCAR), missing data that are at random (MAR), and missing data that are not at random (MNAR) (Little & Rubin, 2002). A special case of missing data not at random is incompleteness because of a *planned missing data design*. Such a design distinguishes between conditions where participants supply data and conditions where participants are not requested to supply data. Planned missing data designs are in use to reduce the costs of empirical research and also to reduce the demands on participants, which is assumed to contribute to the validity of data as a side effect (Rhemtulla & Hancock, 2016; Rioux et al., 2020).

Since most statistical procedures require complete data, methods for compensating for what is missing have been developed. They enable the computation of replacements for what is missing. For example, there is the full information maximum likelihood estimation method that can be employed for this purpose (Enders, 2001). It provides parameter estimates that are used for completing incomplete data sets. Furthermore, there are a number of multiple imputation procedures (Graham, 2009; van Buuren, 2018). They generate plausible replacements for missing data. Besides the replacement methods, there is also a method that seeks to model missing data (Schweizer, Gold, Krampen, & Wang, 2020) that is suitable for factor-analytic investigations of structure. Modeling missing data assumes that incomplete data give rise to two different kinds of systematic variation: systematic variation associated with the measured attribute and systematic variation associated with missing data, that is, systematic variation associated with the frequency distribution of missings is assumed.

The modeling missing data approach proceeds from the conceptualization of latent variables as means to account for the systematic variation of data, as is characteristic of the covariance model that is basic to the maximum likelihood estimation approach of factor analysis (Jöreskog, 1970). In this approach, an individual latent variable accounts for systematic variation which several manifest variables have in common; it is also alternatively said that the latent variable accounts for systematic variation of several manifest variables which is due to the same underlying source. For example, it is assumed that the latent variable of the customary one-factor confirmatory factor analysis (CFA) model (Graham, 2006) accounts for the systematic variation associated with the measured attribute. In the modeling missing data approach, it is additionally assumed that missing data can be transformed into systematic variation of its own (for details see next sections). Systematic variation of its own means that the attribute latent variable cannot capture it. The lack of this latent variable to account for the systematic variation associated with missing data necessitates the consideration of an additional missing data latent variable. This latent variable enlarges the customary one-factor CFA model to also capture the systematic variation associated with missing data. Unlike the other methods, modeling missing data does not require making assumptions on the contributions of systematic and random influences to the responses that are missing.

The research reported in this article is supposed to extend and further explore the modeling missing data approach. Special circumstances have been realized when demonstrating the efficiency of this approach: they consisted of binary data transformed into probability-based covariances that served as input to CFA. Constituents of such covariances are the probabilities of selected response options (e.g., correct responses) while other response options and omissions are ignored. Although missing data exert a systematic influence on the outcome of the computation, they do not need to be explicitly taken into consideration. Therefore, it is an open question whether the modeling missing data approach performs equally well in other types of input to CFA. For example, there are Pearson correlations, polychoric correlations, and tetrachoric correlations that can serve this purpose.

Furthermore, it is not clear which type of factor loadings is likely to lead to valid results. The factor loadings considered in this article introducing the modeling missing data approach were factor loadings fixed according to the relative frequencies of missing data while the possibility to employ free factor loadings was ignored. Fixed factor loadings are also in use in longitudinal research and invariance analyses (McArdle, 2009; McArdle & Cattell, 1994; Schmitt & Kuljanin, 2008). The precondition for using fixed factor loadings is the availability of expectations regarding the relationships among the factor loadings of the manifest variables on the latent variable. In contrast, free factor loadings do not require such expectations. So far, the possibility to use free factor loadings in modeling missing data has not been explored. In investigating the effects of the two types of factor loadings, it needs to be taken into consideration that the two-factor model including a missing data latent variable is the bifactor model (Reise, 2012). As a consequence, it must be assured that not all manifest variables show cross-loadings when using free factor loadings. At least one manifest variable has to be restricted to load on only one of the two latent variables.

In the following, we describe modeling latent variables with tetrachoric correlations as input to CFA in some more detail. Furthermore, a simulation study is reported that employs fixed as well as free factor loadings.

## The Two-Factor Measurement Model for Modeling Missing Data

The modeling missing data approach assumes that missing data cause additional systematic variation besides the systematic variation due to the measured attribute. The customary CFA measurement model includes the attribute latent variable, *ξ*_attribute_, accounting for attribute-related systematic variation. The modeling missing data approach suggests that the additional systematic variation associated with missing data can be captured by a missing data latent variable, *ξ*_missing_. A missing data latent variable accounting for systematic variation due to missing data opens up the possibility to investigate the correctness of a hypothesized structural model despite systematic variation due to missing data. This is possible because the additional systematic variation is removed as potential source of model misfit.

A good start for introducing the measurement model proposed for modeling missing data is provided by the congeneric model (Graham, 2006) that is given by:

(1) $$x=\lambda_{\mathrm{attribute}}\xi_{\mathrm{attribute}}+\delta ,$$

where x is the centered *p*× 1 vector of manifest variables, **λ**_attribute_ the *p*× 1 vector of factor loadings on the attribute latent variable, $\xi_{\mathrm{attribute}}$ , and δ the *p*× 1 vector of error influences. This model accounts for systematic variation associated with the attribute but not for other systematic variation.

For providing a model that also accounts for systematic variation due to missing data, the measurement model has to include another latent variable that targets systematic variation due to missing data. Modifying Equation 1 accordingly leads to:

(2) $$x=\lambda_{\mathrm{attribute}}\xi_{\mathrm{attribute}}+\lambda_{\mathrm{missing}}\xi_{\mathrm{missing}}+\delta .$$

This equation includes an additional component that is the product of the *p*× 1 vector of factor loadings, **λ**_missing_, and the latent variable, *ξ*_missing_. We refer to this model as *missing data model*.

The missing data model is a two-factor CFA model with many cross-loadings on the two latent variables. If one of the two latent variables does not show factor loadings from all manifest variables, this two-factor model is a bifactor model (Reise, 2012). In the case of the fixation of factor loadings, each manifest variable can be linked to each latent variable. Here, only the variance parameters of the latent variables are estimated.

## Tetrachoric Correlations as Input to CFA

The switch from probability-based covariances to tetrachoric correlations as input to CFA means a replacement of one coefficient by another coefficient with differing properties although both are computed from binary data. They differ according to their sizes and the proneness to produce matrices that are not positive definite. Tetrachoric correlations are usually larger than probability-based covariances and, as a consequence, they are more likely to give rise to matrices that are not positive definite.

The probability-based covariance, cov(*X_i_*, *X_j_*), for binary variables *X_i_* and *X_j_* (*i*, *j* = 1, . . ., *p*) is defined as:

(3) $$\mathrm{cov}\left(X_{i},X_{j}\right)=\mathrm{Pr}\left(X_{l}=1\wedge X_{j}=1\right)-\mathrm{Pr}\left(X_{i}=1\right)\mathrm{Pr}\left(X_{j}=1\right),$$

where 1 serves as the code for the correct response. Equation 3 reveals that in a first step the coded binary responses are transformed into probabilities that are considered continuous. Combining the probabilities according to a given scheme (see Equation 3) yields the covariance.

Since the focus is on correct responses, it is not necessary to consider missing data and to distinguish between incorrect responses and missing data. But this does not mean that missing data are without influence on the result. Instead, they exert a systematic influence on the result since they lower the numbers of correct responses. For demonstrating the systematic influence of missing data, we relate the probability of a correct response in item *X_i_*, Pr(*X_i_* = 1), in the incomplete data set to the probability of such a response in the complete data set that only depends on the latent attribute, *ξ*_attribute_, as systematic source, Pr(*X_i_* = 1|*ξ*_attribute_). Because of the incompleteness of the sample, the probability of a correct response also depends on the probability of missing data, Pr(*X_i_* is missing), so that:

(4) $$\mathrm{Pr}{\left(X_{i}=1\right)}_{\mathrm{incomplete}\_\mathrm{data}\;\mathrm{set}}=\mathrm{Pr}{\left(X_{i}=1|\xi_{\mathrm{attribute}}\right)}_{\mathrm{complete}\_\mathrm{data}\;\mathrm{set}}\times \left[1-\mathrm{Pr}\left(X_{i}\;\mathrm{is}\;\mathrm{missing}\right)\right].$$

This means that probabilities contributing to the probability-based covariance include a systematic influence of missing data. This suggests that missing data systematically influence the sizes of covariances (and correlations). They lead to deviations from the sizes that would otherwise be observed. Such deviations in several manifest variables mean systematic variation for which a special latent variable may account.

The tetrachoric correlation is also computed from binary data (Pearson, 1900). In a first step, binary data are transformed into continuous and normally distributed data. This is achieved by latent thresholds, *τ* (Tallis, 1962). With the help of a latent threshold binary random variable *X_i_* is transformed into continuous random variable *V_i_* (*i* = 1, . . ., *p*) following the normal distribution. Threshold *τ* is selected such that the probability that *X_i_* equals one, Pr(*X_i_* = 1), corresponds to the probability that *V_i_* is larger than *τ*:

(5) $$\mathrm{Pr}\left(X_{i}=1\right)=\mathrm{Pr}\left(V_{i}>\tau \right).$$

Subsequently, the relationship of variables *V_i_* and *V_j_* (*i, j* = 1, . . ., *p, i ≠ j*), that is, the tetrachoric correlation of *V_i_* and *V_j_*, is estimated.

Programs for estimating tetrachoric correlations require complete data (e.g., Prelis 2 by Jöreskog & Sörbom, 1999). This means that missing data cannot be ignored but need to be replaced. Software for preparing the input to factor analysis provides options for automatically replacing missing data. An alternative point of view is that replacing missing data by zeros (code for incorrect response) creates the same situation as with probability-based covariances: missings influence the probabilities of Equation 5 in the same way as is outlined in Equation 4 so that an effect of missing data can be expected.

## The Two Types of Factor Loadings

Factor loadings can be fixed or set free for estimation whereby the latter option is the more common case. Free factor loadings are easy to realize. In the case of CFA with two overlapping factors and free factor loadings, it is important that one factor loading is fixed to zero, as is required for the bifactor model (Reise, 2012). But this restriction only holds if all columns of a data set show missing data. All factor loadings of **λ**_attribute_ (see Equation 2) have to be set free for estimation, whereas one of the factor loadings of **λ**_missing_ needs to be fixed. In Equation 6, it is the first factor loading that is fixed to zero:

(6) $$\lambda_{\mathrm{missing}}=\left[\begin{array}{c} 0\\ \lambda_{\mathrm{missing}\_2}\\ .\\ .\\ \lambda_{\mathrm{missing}\_p} \end{array}\right].$$

where *λ*_missing_2_ to *λ*_missing_*p*_ are free factor loadings.

Selecting fixed instead of free factor loadings means the assignment of numbers to the slots of the vector of factor loadings. The numbers should be related to the frequencies of missing data in the corresponding columns of the data set that is under investigation. In a planned missing data design, the information on the frequency distribution of missing data is available from the design. For each column *i* (*i* = 1, . . ., *p*) of the data set the expected number of missing data, *n_i_*, can be ascertained from the design. Since only the information on the relationships among the frequencies of missings is of importance, adjusting the frequencies to the typical sizes of factor loadings is a modification that does not influence the outcome regarding model fit but can support the estimation process. If all columns with missing data show the same number of missings, it is sufficient to assign the same constant *c* (>0) to the corresponding slots of the vector of factor loadings. To have an example, we assume that the first half of the columns of the data set show no missings and the other half the same numbers of missings. In this case, the following *p*× 1 vector of factor loadings, **λ**_missing_, is suitable for the investigation:

(7) $$\lambda_{\mathrm{missing}}=\left[\begin{array}{c} 0\\ .\\ .\\ 0\\ c\\ .\\ .\\ c \end{array}\right].$$

A selection that is perfectly suitable for this purpose is *c* = 1. The sizes of numbers used as factor loadings do usually not influence model fit but the estimate of the variance parameter. If variance estimates are to be used for the purposes of interpretation or comparison, they must be scaled appropriately (Schweizer et al., 2019). What is special of missing data analysis with fixed factor loadings compared with missing data analysis with free factor loadings is that there is no need to fix one factor loading to zero.

The fixation of the entries of **λ**_missing_ as, for example, in Equation 6 necessitates the freeing of the associated variance parameter, *φ*_missing_, of the model of the *p*×*p* covariance matrix, **Σ**, for estimation. Estimation is accomplished as minimization of the difference between **Σ** specified by parameter estimates that are represented by *θ* and the *p*×*p* empirical covariance matrix, **S**, using discrepancy function F:

(8) F[Σ(θ),S].

The latent variable, *ξ*_missing_, accounts for the systematic variation due to missing data. Depending on what is fixed and what is set free, either the estimate of *φ*_missing_ or the estimates of **λ**_missing_ reflect the amount of systematic variation captured by *ξ*_missing_.

The fixation of the factor loadings of **λ**_missing_ on *ξ*_missing_ does not imply any restriction to the factor loadings of **λ**_attribute_ on *ξ*_attribute_. This means that the factor loadings included in **λ**_attribute_ can be defined as free or fixed parameters so that even hybrid measurement models are possible.

## A Simulation Study on Modeling Missing Data

The first major aim of the simulation study was the demonstration that modeling missing data can be conducted with tetrachoric correlations as input to CFA. Modeling of missing data was realized as an additional missing data latent variable integrated into the regular CFA measurement model. Tetrachoric correlations computed from binary data that were either complete or incomplete were investigated. The binary data showed a unidimensional latent structure that was to be demonstrated. The second major aim was to investigate the consequences of selecting either fixed or free factor loadings for modeling missing data. The investigation focused on model fit and mainly consisted in comparisons of results obtained by CFA models with and without a missing data latent variable. The performance of the one-factor CFA model served as comparison level.

### Method

Three-hundred 500 × 20 matrices of normally distributed random data were generated, transformed into binary data (0 and 1) and modified to show different missing data conditions. The latent unidimensional structure was achieved by the procedure described by Jöreskog and Sörbom (2001). It required the preparation of a relational pattern. We selected 1.00 for the diagonal entries of this relational pattern and the square of 0.35 for the off-diagonal entries so that the expected factor loadings for the one-factor CFA model were 0.35. We selected a value as small as 0.35 because it created a condition that allowed for good as well as bad model fit in structural investigations.

Missing data were realized in the following way: we eliminated data from either four (columns 17–20) or 10 (columns 11–20) of the total of 20 columns of the generated data sets. Furthermore, the percentages of eliminated entries were varied. Based on previous experiences, either 20%, 40%, or 60% of the entries of the selected columns were removed. Although the rows for eliminating entries were selected according to a quasi-random scheme, the columns selected for the elimination of entries were kept constant.

Tetrachoric correlations served as input to CFA. Such correlations were computed before and after inserting missing data into the data sets by means of Prelis (Jöreskog & Sörbom, 2001). Since Prelis expected complete data, missings were replaced by zeros (for justification see last paragraph of section titled “tetrachoric correlations as input to CFA”). Zeros were expected to provide the basis for systematic variation due to missing data.

The structure of data was investigated by one-factor and two-factor models with 20 manifest variables. One-factor models served the investigation of complete and incomplete data, whereas two-factor models were only used for the investigation of incomplete data. There were versions with free and fixed factor loadings.

In the case of fixed factor loadings, the sizes of all factor loadings of the manifest variables on the attribute latent variable were set equal to one since in generating data it was assumed that the latent variable contributed equally to all columns of a data set; that is, all manifest variables. The sizes of the factor loadings on the missing data latent variable were set to one for columns with missing data and to zero otherwise. The variance parameters of the attribute latent variable and the missing data latent variable were set free for estimation.

All factor loadings were set free for estimation when using free factor loadings for the attribute latent variable. The factor loadings on the missing data latent variable associated with columns of data sets showing missing data were set free while all other ones were set to zero. The variance parameters of the attribute latent variable and the missing data latent variable were fixed to one. A note based on experiences: Setting all factor loadings or all factor loadings minus one on the missing data latent variable free did not work out well. Further advice is provided by an practice example (see Appendix).

The LISREL software package (Jöreskog & Sörbom, 2006) with Satorra-Bentler corrected maximum likelihood estimation was used. We employed the following fit indices and established criteria (in parentheses) for this study: χ^2^, root mean square error of approximation (RMSEA) (≤0.06), standardized root mean square residual (SRMR) (≤0.08), nonnormed fit index (NNFI) (≥0.95), and comparative fit index (CFI) (≥0.95) (see DiStefano, 2016; Hu & Bentler, 1999). The CFI difference (0.01) and RMSEA difference (0.015) criteria according to Cheung and Rensvold (2002) were used to compare models.

## Results

In the following, the results obtained by models with free and fixed factor loadings are reported separately. The interpretation concentrates on fit indices with a cut-off (RMSEA, SRMR, NNFI, CFI).

### Results Obtained by Customary Models With Fixed Factor Loadings

Table 1 includes the mean fit results and confidence intervals (*in italics*) observed in investigations based on tetrachoric correlations that were computed from data sets with and without missing data by the customary one-factor CFA model with fixed factor loadings.

**Table 1:** Mean Fit Results and 95% Confidence Intervals Observed in Investigations Without and With Missing Data by the Customary One-Factor Model with Fixed Factor Loadings (N = 300)

Model	Missing per column/overall	χ^2^	*df*	RMSEA	SRMR	NNFI	CFI	AIC
One factor	0%/0%	200.5	189	0.009	0.087	0.986	0.982	242.5
		*[196.8*, *204.2]*		*[0.008*, *0.010]*	*[0.086*, *0.088]*	*[0.979*, *0.993]*	*[0.975*, *0.989]*	*[238.8*, *246.2]*
Missing data in four columns								
One factor	20%/4%	260.6	189	0.027	0.098	0.952	0.952	302.6
		*[256.3*, *264.9]*		*[0.026*, *0.028]*	*[0.097*, *0.099]*	*[0.943*, *0.961]*	*[0.943*, *0.961]*	*[298.2*, *306.9]*
One factor	40%/8%	378.8	189	0.045	0.118	0.906	0.906	420.8
		*[374.1*, *383.5]*		*[0.045*, *0.046]*	*[0.116*, *0.120]*	*[0.895*, *0.917]*	*[0.895*, *0.917]*	*[416.1*, *425.5]*
One factor	60%/12%	500.1	189	0.057	0.135	0.856	0.857	542.1
		*[499.6*, *500.6]*		*[0.057*, *0.057]*	*[0.135*, *0.135]*	*[0.855*, *0.857]*	*[0.856*, *0.858]*	*[541.6*, *542.6]*
Missing data in 10 columns								
One factor	20%/10%	591.1	189	0.065	0.178	0.917	0.917	633.1
		*[582.8*, *599.4]*		*[0.064*, *0.066]*	*[0.176*, *0.180]*	*[0.909*, *0.925]*	*[0.909*, *0.925]*	*[624.8*, *641.4]*
One factor	40%/20%	1,138.6	189	0.100	0.267	0.884	0.885	1,180.6
		*[1,124.9*, *1,152.2]*		*[0.099*, *0.101]*	*[0.263*, *0.271]*	*[0.873*, *0.895]*	*[0.875*, *0.895]*	*[1,166.9*, *1,194.3]*
One factor	60%/30%	1,315.4	189	0.109	0.289	0.863	0.864	1,357.4
		*[1,286.6*, *1,344.2]*		*[0.108*, *0.110]*	*[0.283*, *0.295]*	*[0.849*, *0.877]*	*[0.850*, *0.878]*	*[1,328.6*, *1,386.2]*

*Note.*χ^2^ = chi-square; *df* = degree of freedom; RMSEA = root mean square error of approximation; SRMR = standardized root mean square residual; NNFI = nonnormed fit index; CFI = comparative fit index; AIC = Akaike information criterion.

The first row of results was achieved by the one-factor CFA model in investigating complete data. RMSEA, NNFI, and CFI indicated good model fit. They signified that the one-factor CFA model fitted to the data in the absence of missings but not SRMR. Results obtained by investigating data sets showing missing data in four columns are included in the second to fourth rows of the table. In these data sets, the overall percentage of missing data varied between 4% and 12%. In data with 4% of missings (second row) good model fit was indicated by RMSEA, NNFI, and CFI while the model fit was not good according to SRMR. The other percentages of missing data led to decreasing model fit. SRMR, NNFI, and CFI indicated bad model fit while RMSEA was still good. Finally, there are the results reported in the fifth to seventh rows. They were obtained by investigating data sets with missing data in 10 columns. All fit indices included in these rows indicated bad model fit according to the cut-offs.

The comparisons of RMSEA and CFI obtained for complete data with RMSEAs and CFIs obtained for incomplete data yielded substantial RMSEA differences in all cases and substantial CFI differences in all but one case. Furthermore, comparisons suggested that increasing the percentage of missing data resulted in a decrease of model fit with one exception.

In sum, the investigation of the effect of the missing data manipulation revealed the expected outcome in all cases but one. The increase of the amount of missing data led to a decrease in model fit.

### Results Obtained by Missing Data Models With Fixed Factor Loadings

The mean fit statistics and confidence intervals (*in italics*) presented in Table 2 were observed in investigating data sets with missing data by missing data models using fixed factor loadings with the exception of the results reported in the first row.

**Table 2:** Mean Fit Results and 95% Confidence Intervals Observed in Investigations Without and With Missing Data by the Missing Data Two-Factor Model With Fixed Factor Loadings (N = 300)

Model	Missing per column/overall	χ^2^	*df*	RMSEA	SRMR	NNFI	CFI	AIC
One factor	0%/0%	200.5	189	0.009	0.087	0.986	0.982	242.5
		*[196.8*, *204.2]*		*[0.008*, *0.010]*	*[0.086*, *0.088]*	*[0.979*, *0.993]*	*[0.975*, *0.989]*	*[238.8*, *246.2]*
Missing data in four columns								
Two factors	20%/4%	200.1	188	0.009	0.087	0.984	0.982	244.1
		*[196.1*, *204.1]*		*[0.008*, *0.010]*	*[0.086*, *0.088]*	*[0.977*, *0.991]*	*[0.976*, *0.988]*	*[240.1*, *248.1]*
Two factors	40%/8%	202.1	188	0.010	0.089	0.986	0.984	246.1
		*[198.1*, *206.1]*		*[0.009*, *0.011]*	*[0.088*, *0.090]*	*[0.981*, *0.991]*	*[0.979*, *0.989]*	*[242.1*, *250.1]*
Two factors	60%/12%	206.6	188	0.011	0.090	0.981	0.979	250.6
		*[206.1*, *207.1]*		*[0.011*, *0.011]*	*[0.090*, *0.090]*	*[0.980*, *0.982]*	*[0.978*, *0.980]*	*[250.1*, *251.1]*
Missing data in 10 columns								
Two factors	20%/10%	203.9	188	0.011	0.088	0.994	0.993	247.9
		*[200.0*, *207.8]*		*[0.010*, *0.012]*	*[0.087*, *0.089]*	*[0.992*, *0.996]*	*[0.991*, *0.995]*	*[244.0*, *251.8]*
Two factors	40%/20%	206.2	188	0.011	0.091	0.996	0.995	250.2
		*[202.0*, *210.4]*		*[0.010*, *0.012]*	*[0.090*, *0.092]*	*[0.995*, *0.997]*	*[0.994*, *0.996]*	*[246.0*, *254.4]*
Two factors	60%/30%	209.9	188	0.012	0.090	0.995	0.995	253.9
		*[204.6*, *215.2]*		*[0.011*, *0.013]*	*[0.088*, *0.092]*	*[0.994*, *0.996]*	*[0.994*, *0.996]*	*[248.6*, *259.2]*

*Note.*χ^2^ = chi-square; *df* = degree of freedom; RMSEA = root mean square error of approximation; SRMR = standardized root mean square residual; NNFI = nonnormed fit index; CFI = comparative fit index; AIC = Akaike information criterion.

The first row of results was adopted from Table 1 to have a comparison level. The results of the second to fourth rows were obtained by investigating data sets with missing data in four columns that amounted to between 4% and 12% missings. All RMSEAs, NNFIs, and CFIs suggested good model fit while SRMRs missed the cut-off by a small amount. Investigating data with missings in 10 columns of the data sets that amounted to between 10% and 30% of missing data led to the results of the fifth to seventh rows. Again, good model fit was signified by all RMSEAs, NNFIs and CFIs while the cut-off for SRMR was missed. The increase of the percentage of missing data did neither influence RMSEA nor CFI in data sets with missing data in four or 10 columns.

The comparisons of RMSEA and CFI obtained for complete data on one hand and of RMSEAs and CFIs obtained for incomplete data on the other hand showed *numeric* improvement associated with missing data. Yet, none of the RMSEA differences was substantial while almost all CFI differences with missing data in 10 columns indicated a substantial improvement in model fit.

In sum, considering a missing data latent variable as part of a CFA model with fixed factor loadings not only prevented impairment in model fit due to missing data but even increased model fit according to CFI.

### Results Obtained by Customary Models With Free Factor Loadings

Table 3 comprises the mean fit results and confidence intervals (*in italics*) observed when investigating structure on the basis of tetrachoric correlations of data with and without missings by the customary one-factor CFA model with free factor loadings.

**Table 3:** Mean Fit Results and 95% Confidence Intervals Observed in Investigations Without and With Missing Data by the Customary One-Factor Model with Free Factor Loadings (N = 300)

Model	Missing per column/overall	χ^2^	*df*	RMSEA	SRMR	NNFI	CFI	AIC
One factor	0%/0%	186.4	170	0.010	0.079	0.981	0.980	266.4
		*[180.3*, *192.5]*		*[0.009*, *0.011]*	*[0.078*, *0.080]*	*[0.973*, *0.989]*	*[0.973*, *0.987]*	*[260.3*, *272.5]*
Missing data in four columns								
One factor	20%/4%	240.3	170	0.027	0.088	0.946	0.951	320.3
		*[232.8*, *240.8]*		*[0.026*, *0.028]*	*[0.087*, *0.089]*	*[0.934*, *0.958]*	*[0.940*, *0.962]*	*[312.8*, *327.8]*
One factor	40%/8%	307.0	170	0.039	0.105	0.924	0.934	387.0
		*[299.5*, *314.5]*		*[0.038*, *0.040]*	*[0.103*, *0.107]*	*[0.913*, *0.935]*	*[0.924*, *0.944]*	*[379.8*, *394.2]*
One factor	60%/12%	320.1	170	0.041	0.110	0.909	0.919	404.1
		*[319.3*, *320.9]*		*[0.041*, *0.041]*	*[0.010*, *0.010]*	*[0.908*, *0.910]*	*[0.918*, *0.920]*	*[403.3*, *404.9]*
Missing data in 10 columns								
One factor	20%/10%	229.9	170	0.023	0.087	0.985	0.986	309.9
		*[217.1*, *242.7]*		*[0.022*, *0.024]*	*[0.085*, *0.089]*	*[0.981*, *0.989]*	*[0.983*, *0.989]*	*[297.1*, *322.7]*
One factor	40%/20%	236.8	170	0.026	0.072	0.986	0.988	316.8
		*[232.0*, *241.6]*		*[0.025*, *0.027]*	*[0.069*, *0.075]*	*[0.984*, *0.988]*	*[0.986*, *0.990]*	*[312.0*, *321.6]*
One factor	60%/30%	225.9	170	0.023	0.085	0.990	0.991	305.9
		*[219.6*, *232.2]*		*[0.022*, *0.024]*	*[0.082*, *0.088]*	*[0.988*, *0.992]*	*[0.989*, *0.993]*	*[299.6*, *312.2]*

*Note.*χ^2^ = chi-square; *df* = degree of freedom; RMSEA = root mean square error of approximation; SRMR = standardized root mean square residual; NNFI = nonnormed fit index; CFI = comparative fit index; AIC = Akaike information criterion.

The results of investigating complete data are provided in the first row. They signified overall good model fit (see RMSEA, SRMR, NNFI and CFI). The results of the second to fourth rows were achieved in investigating data sets showing four columns with missing data. In these data sets, the percentage of missing data varied between 4% and 12%. All RMSEAs and one of the three CFIs indicated good model fit. No SRMR or NNFI reached the cut-off for good model fit. A different pattern of results characterized the data sets with missing data in 10 columns, as is apparent from the fifth to seventh rows. Despite missing data, good model fit was signified by all RMSEAs, NNFIs, CFIs and one of the three SRMRs. The remaining SRMRs missed the cut-off by a small amount.

Comparing RMSEA and CFI obtained for complete data with RMSEAs and CFIs obtained for incomplete data yielded mixed results. In data sets with four columns including missing data, substantial RMSEA differences were observed whereas only one substantial RMSEA differences were found when the number of columns with missing data was 10. Similarly, CFI differences suggested impairment in model fit for data sets with four columns including missing data and improvement in model fit when data sets with 10 columns included missing data.

In sum, the effect of the missing data manipulation depended on the number of columns with missing data when the model included free factor loadings. Missing data in four columns of a data set led to a decrease in model fit whereas there was constancy or increase in model fit when the number of columns including missing data was 10.

### Results Obtained by Missing Data Models With Free Factor Loadings

Investigations of data sets with missing data by missing data models including free factor loadings led to the mean fit statistics and confidence intervals (*in italics*) presented in Table 4 with the exception of the results reported in the first row.

**Table 4:** Mean Fit Results and 95% Confidence Intervals Observed in Investigations Without and With Missing Data by the Missing Data Two-Factor Model with Free Factor Loadings (N = 300)

Model	Missing per column/overall	χ^2^	*df*	RMSEA	SRMR	NNFI	CFI	AIC
One factor	0%/0%	186.4	170	0.010	0.079	0.981	0.980	266.4
		*[180.3*, *192.5]*		*[0.009*, *0.011]*	*[0.078*, *0.080]*	*[0.973*, *0.989]*	*[0.973*, *0.987]*	*[260.3*, *272.5]*
Missing data in four columns								
Two factors	20%/10%	177.8	160	0.010	0.077	0.983	0.982	265.8
		*[173.9*, *181.7]*		*[0.009*, *0.011]*	*[0.076*, *0.078]*	*[0.976*, *0.990]*	*[0.976*, *0.988]*	*[261.9*, *269.7]*
Two factors	40%/20%	179.1	160	0.010	0.078	0.985	0.985	267.1
		*[175.2*, *183.0]*		*[0.009*, *0.011]*	*[0.077*, *0.079]*	*[0.979*, *0.991]*	*[0.980*, *0.990]*	*[263.2*, *271.0]*
Two factors	60%/30%	184.2	160	0.011	0.079	0.980	0.980	272.2
		*[183.8*, *184.6]*		*[0.011*, *0.011]*	*[0.079*, *0.079]*	*[0.979*, *0.981]*	*[0.979*, *0.981]*	*[271.8*, *272.6]*
Missing data in 10 columns								
Two factors	20%/10%	170.8	160	0.010	0.078	0.995	0.995	270.8
		*[167.4*, *174.2]*		*[0.009*, *0.011*	*[0.076*, *0.080]*	*[0.993*, *0.997]*	*[0.993*, *0.997]*	*[267.4*, *274.2]*
Two factors	40%/20%	170.2	160	0.010	0.081	0.997	0.997	270.2
		*[166.8*, *173.6]*		*[0.009*, *0.011]*	*[0.079*, *0.083]*	*[0.996*, *0.998]*	*[0.996*, *0.998]*	*[266.8*, *273.6]*
Two factors	60%/30%	172.1	160	0.010	0.082	0.997	0.997	272.1
		*[168.4*, *175.8]*		*[0.009*, *0.011]*	*[0.080*, *0.084]*	*[0.996*, *0.998]*	*[0.996*, *0.998]*	*[268.4*, *275.8]*

*Note.*χ^2^ = chi-square; *df* = degree of freedom; RMSEA = root mean square error of approximation; SRMR = standardized root mean square residual; NNFI = nonnormed fit index; CFI = comparative fit index; AIC = Akaike information criterion.

The investigation of data with missings in four columns yielded good model fit according to RMSEA, SRMR, NNFI, and CFI (see second to fourth rows). When the number of columns with missing data was 10, good model fit was indicated by RMSEA, NNFI, and CFI. In one of the three cases, SRMR was also good.

The comparisons of RMSEA and CFI obtained for complete data and RMSEAs and CFIs obtained for incomplete data led to different results for RMSEA and CFI. All RMSEA differences suggested constancy, that is, no difference between the results for complete data and incomplete data. In contrast, according to all CFI differences, model fit was better in incomplete data than in complete data when the number of columns with missing data was 10.

In sum, considering a missing data latent variable as part of a CFA model with free factor loadings assured good model fit. But, this might not mean recovery of the model fit characterizing complete data since not all results for data sets with missing data showed impairment in model fit when investigated by the one-factor CFA model.

### Results Regarding the Major Aims

In this section, we summarize the results regarding the two major aims based on the comparisons by RMSEA and CFI differences. We provide percentages of correctly detected impairments of model fit due to the presence of missing data and percentages of correctly recovered original levels of model fit due to the use of a measurement model including a missing data latent variable. Sensitivity to missing data is illustrated by bars in the negative direction and recovery to the original level by bars in the positive direction. In all cases, 100% means that all mean results are according to expectations.

**Figure 1:**
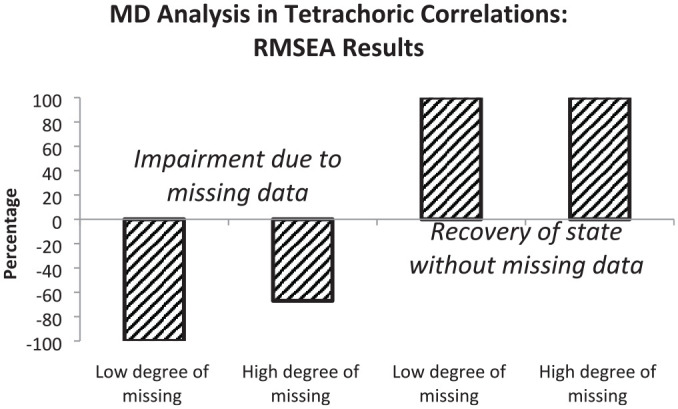
Graphical Representation of Percentages of RMSEA Results Indicating Impairment in Model Fit (First and Second Bars) or Restoration of Fit Level Without Missing Data (Third and Fourth Bars) Observed in Missing Data Analysis (MD Analysis) Based on Tetrachoric Correlations *Note.* Oblique hatching signifies results within expected range. RMSEA = root mean square error of approximation.

The first major aim of the simulation study was the demonstration of the efficiency of modeling missing data with tetrachoric correlations as input. Figure 1 provides the percentages based on RMSEA differences.

**Figure 2:**
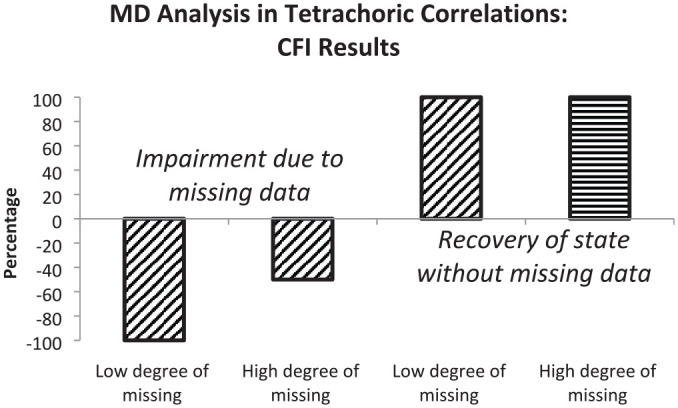
Graphical Representation of Percentages of CFI Results Indicating Impairment in Model Fit (First and Second Bars) or Restoration of Fit Level Without Missing Data (Third and Fourth Bars) Observed in Missing Data Analysis (MD Analysis) Based on Tetrachoric Correlations *Note.* Oblique hatching signifies results within expected range and horizontal hatching results beyond the expected range. CFI = comparative fit index.

According to the first and second bars that illustrate RMSEA results, the sensitivity for missing data depended on the amount of missings. In data with a small amount of missing data (between 4% and 12%), the sensitivity for impairment was higher than in data with a large amount of missing data (between 10% and 30%). In both types of data, there was full recovery of the model fit characterizing complete data (see third and fourth bars). Figure 2 illustrates the percentages based on CFI differences.

The first bar signifies a high degree of sensitivity for missing data when there was a small amount of missing data and the second bar a medium degree of sensitivity when there was a large amount of missing data. The sizes of the third and fourth bars signify full recovery of the level of model fit characterizing complete data when considering a missing data latent variable. The horizontal hatching of the fourth bar additionally indicates that there was not only recovery but also improvement of model fit over the original level.

**Figure 3:**
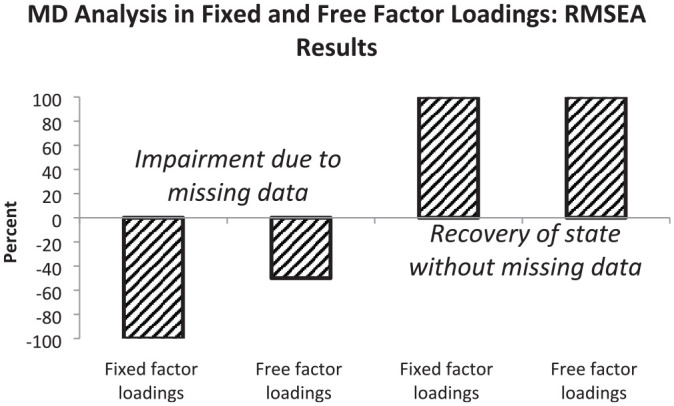
Graphical Representation of Percentages of RMSEA Results Indicating Impairment in Model Fit (First and Second Bars) or Restoration of Fit Level Without Missing Data (Third and Fourth Bars) Observed by Measurement Models With Fixed and Free Factor Loadings *Note.* Oblique hatching signifies results within expected. RMSEA = root mean square error of approximation.

The second major aim of the simulation study was the comparison of the effects of fixed and free factor loadings regarding their efficiency in modeling missing data. The percentages based on RMSEA differences are illustrated by Figure 3.

**Figure 4:**
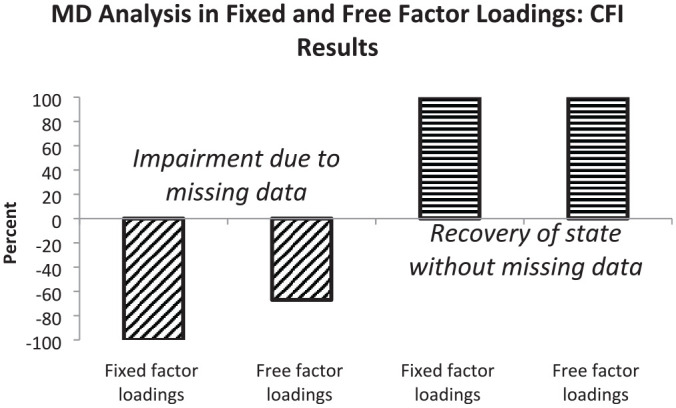
Graphical Representation of Percentages of CFI Results Indicating Impairment in Model Fit (First and Second Bars) or Restoration of Fit Level Without Missing Data (Third and Fourth Bars) Observed by Measurement Models With Fixed and Free Factor Loadings *Note.* Oblique hatching signifies results within expected range and horizontal hatching results beyond the expected range. CFI = comparative fit index.

When the measurement model included fixed factor loadings, the sensitivity for missing data was high (see first bar). The inclusion of free factor loadings into the measurement model was associated with a medium degree of sensitivity for missing data only (see second bar). Full recovery of model fit for complete data was observed for measurement models with fixed and free factor loadings (see third and fourth bars). Figure 4 depicts the percentages based on CFI differences regarding the effects of fixed and free factor loadings regarding their efficiency in modeling missing data.

The first bar indicates that in using fixed factor loadings the sensitivity for missing data was high while the second bar only signifies a medium degree of sensitivity for free factor loadings. According to the third and fourth bars, an additional missing data latent variable led not only to the recovery of the level of model fit characterizing data without missings but also to an increase in model fit (see hatching), especially when there was a large amount of missing data.

## Discussion

Statistical methods for investigating empirical data usually require completeness. But, the truth is that empirical data are often incomplete, that is, responses of some participants of the sample are missing. Since in collecting empirical data mostly multiple responses are required, that is, a participant has to provide more than just one response, participants providing no response at all are very rare. The more common case is that participants provide at least some responses so that participants with missing responses are in a way known and can even be characterized by the few responses that are available (Vogt & Johnson, 2015). But, what never will be known is what would have been the responses that did not occur or find their way into the data set.

Methods have been developed for the replacement of missing data to enable the statistical investigation of data. For example, the maximum likelihood framework that is used for estimating model parameters is also employed for estimating missing responses (Enders, 2001). The focus is on providing estimates of what characterizes the participants whose responses are missing. Furthermore, so-called multiple imputation methods for creating values for replacing missing responses have been developed (van Buuren, 2018). These methods attempt to additionally take uncertainty due to random influences into consideration. Multiple samples with replacements are created and combined for obtaining values reflecting all kinds of influences, systematic and random ones, on a possible response.

However, including numbers as replacements for missings into a data set may not be without consequences for this data set. Replacements can increase the consistency among some or all items of a scale and, thus, it can increase the probability of detecting something that appears to underlie the data but can also contribute to inhomogeneity. If a data set includes a larger percentage of missing data, replacements can enlarge the consistency among the columns of a data set and increase the factor loadings that are crucial in detecting an underlying dimension in a factor-analytic investigation (Widaman, 2018). Replacements can lead to fit results that create the impression of an underlying dimension in data where otherwise no such dimension would be detected. Furthermore, missing data influence the retention of the number of latent variables in explorative investigations (Goretzko, 2022).

The avoidance of replacements of missings is a way of preventing any kind of influence on the properties of an incomplete data set (Schweizer, Gold, Krampen, & Wang, 2020). This approach of dealing with missing data assumes that missing data give rise to systematic variation that can be captured by a latent variable in factor analysis. It fits especially well with research designs assuring that data sets show larger coherent chunks of missing data so that there is a solid basis for a missing data latent variable. Such a condition characterizes data that are collected according to a planned missing data designs (Rhemtulla & Hancock, 2016; Rioux et al., 2020). A planned missing data design is an incomplete design. Starting from a complete design, those design parts are eliminated that are not really necessary for achieving valid results regarding core questions of a research project. In an investigation making use of such a design, the main focus is usually on effects and on relationships between constructs. Although the structural properties of data may be of secondary importance only, it can still be desirable to demonstrate assumed underlying dimensions.

One major reason for conducting the reported study was to extend the modeling missing data approach to tetrachoric correlations as input to factor analysis since this approach was introduced on the basis of probability-based covariances. Although tetrachoric correlations are also computed from binary data, they usually show a larger size and frequently lead to correlation matrices that are not positive definite. This often initiates the automatic call of a ridge option that modifies the input to factor analysis (Yuan et al., 2011) to arrive at a positive definite correlation matrix. The results of our study reveal that the switch to tetrachoric correlations as input to factor analysis does not impair the efficiency of modeling missing data. In all conditions of the design of the study, the additional missing data latent variable led to fit levels of data sets with missings that corresponded to the fit levels characterizing complete data sets or even to an improvement over these levels.

Improvements are not expected and, therefore, may be considered as invalid outcomes. But, there is a rational explanation for the observation of improvements: including missing data into a complete data set can mean a general reduction of the degree of heterogeneity because eliminating data from a data set means not only elimination of systematic variation but also random variation. After removing data from a data set there is usually no more as much random variation as before. This may especially influence fit indices that to some degree reflect such random variation, as appears to be the case of CFI (Bentler, 1990). CFI seems to be sensitive to the elimination of heterogeneity due to random influences.

Other deviations from expectations are regarding SRMR and Akaike information criterion (AIC). Whereas RMSEA, CFI, and NNFI results indicate good model fit when the CFA model is appropriate for missing data, SRMR signifies model misfit. The probable reason for this “odd” behavior is Satorra-Bentler correction. This correction leads to a post hoc modification of χ^2^ in the first place but there are also consequences for some other fit indices. This is because χ^2^ is an ingredient of many fit indices but not of SRMR. This means that the other fit indices of the study undergo a correction that results into a change to a more positive outcome whereas SRMR stays the same. In the case of AIC there is consistent dependency on the sample size that cannot be found in the other fit indices except of χ^2^. AIC shows an especially strong dependency on χ^2^.

The other major aim was to find out whether similar results are obtainable by models with fixed and free factor loadings. The first study testing the modeling missing data approach only included models with fixed factor loadings so that it was an open question whether the results could be generalized to free factor loadings. Now there are results for providing an answer to this question. The missing data latent variable showed to be equally efficient in combination with fixed and free factor loadings. In both cases, it led to fit levels characterizing data sets with missing data that largely corresponded to the fit levels characterizing complete data sets. Furthermore, there were improvements over these levels (a previous paragraph provides an explanation for improvements). What differed between the two types of factor loadings was the performance of the one-factor CFA model in data with missings. In models with fixed factor loadings there was virtually always the expected decrease in model fit due to missing data whereas in models with free factor loadings there were mixed results. In some conditions, a decrease in model fit was observed whereas in other conditions there was no indication of a decrease.

The fit results revealing a difference between models with fixed factor loadings and models with free factor loadings seem to suggest different ways of functioning. Models with fixed factor loadings turn out to be sensitive to missing data. One-factor CFA models with fixed factor loadings show an impairment in model fit that seems to reflect the amount of missing data. The impairment is reversed after including a missing data latent variable into the model. This suggests the interpretation that the missing data latent variable accounts for the systematic variation associated with missing data. In contrast, models with free factor loadings show a reduced sensitivity for missing data. There is not the kind of systematic impairment that characterizes fixed factor loadings. Therefore, it is less obvious in the case of free factor loadings whether there is recovery of the original model fit by compensating for the effect of missing data or just another comprehensive representation.

There is a difference between this study on modeling missing data and the study introducing modeling missing data that needs to be pointed out. The previous study included two types of missing CFA models whereas in this study only one of the two types is realized. The omitted type of model is the semi-hierarchical model (Schweizer, Gold, & Krampen, 2020). The semi-hierarchical model takes the hierarchical structure of data into consideration by means of weights. It showed the better model fit under virtually all conditions and contributed to the correct recovery of factor loadings. This model is not included in the present study because LISREL does not allow assigning weights to free factor loadings. Otherwise, models with fixed factor loadings would be realized as semi-hierarchical models and models with free factor loadings as customary models.

There are some limitations of the reported study. First, only binary data were considered in generating the input to factor analysis. Second, the size of the investigated data sets was kept constant. Especially, it is an open question whether the models perform equally well in small numbers of manifest variables as in the number selected for the study. Third, there is no variation of the expected value of the factor loadings.

Despite these limitations, the study demonstrates that modeling missing data yields fit results in structural investigations of incomplete data with tetrachoric correlations as input corresponding to fit results for complete data. While models with fixed and free factor loadings appear to do equally well in restoring the level of model fit characterizing complete data, models with fixed factor loadings display the better sensitivity for missing data. This method for dealing with missing data is available for data collected according to a planned missing data design and other conditions giving rise to a systematic loss of data in data collection (e.g., ethical concerns).

## Appendix

A data matrix showing the same properties as the matrices of the reported study except of that it included only 10 columns (500 × 10 matrix) was prepared (MMD_examplc.txt [“outset_matrix”]). Afterwards 50% of the entries were removed from Columns 6 to 10. Subsequently, the missings were replaced by zeros for obtaining the final matrix (MMD_examplm.txt [“missing_data_matrix”]).

### The Input

The computation of tetrachoric correlations for the outset matrix by means of PRELIS (an addendum to software package LISREL) led to the following correlation matrix:

**Table:**

	I1	I2	I3	I4	I5	I6	I7	I8	I9	I10
I1	1.000									
I2	.349	1.000								
I3	.095	.171	1.000							
I4	.178	.060	.101	1.000						
I5	.056	.001	.249	.096	1.000					
I6	.136	.311	.130	.048	.007	1.000				
I7	.139	.082	.124	.114	.286	.127	1.000			
I8	–.038	.082	.314	.090	.013	.016	.101	1.000		
I9	.224	.060	.238	.089	.120	.028	–.026	.158	1.000	
I10	.126	.286	.120	.034	.161	–.007	.180	.174	.151	1.000

and for the missing data matrix to the next one:

**Table:**

	I1	I2	I3	I4	I5	I6	I7	I8	I9	I10
I1	1.000									
I2	.349	1.000								
I3	.095	.171	1.000							
I4	.178	.060	.101	1.000						
I5	.056	.001	.249	.096	1.000					
I6	.112	.200	–.041	.082	–.011	1.000				
I7	.308	–.017	.016	.206	.170	.763	1.000			
I8	.052	–.013	.059	.076	.032	.748	.743	1.000		
I9	.204	–.027	.085	.152	.068	.708	.674	.681	1.000	
I10	.196	.413	.000	.084	.126	.639	.699	.665	.638	1.000

PRELIS also produces an asymptotic covariance matrix that is necessary for the Satorra-Bentler correction. This matrix is not accessible to the user and, therefore, cannot be provided.

### LISREL Code for Fixed Factor Loadings

Program for one-factor model with fixed factor loadings

DA NI=10 NO=500 MA=KM

LA

I1 I2 I3 I4 I5 I6 I7 I8 I9 I10

KM FI=outset_matrix.cor ! tetrachoric correlations

AC FI=outset_matrix.acc ! asymptotic covariance matrix

MO NX=10 NK=1 TD=DI, FR PH=FU, FI

LK

Main_factor

VA 1 LX 1 1

. . . . . . . . . . .

VA 1 LX 10 1

FR PH 1 1

OU ML SC IT=1000 AD=OFF ND=3

Program for two-factor model with fixed factor loadings

DA NI=10 NO=500 MA=KM

LA

I1 I2 I3 I4 I5 I6 I7 I8 I9 I10

KM FI=missing_data_matrix.cor ! tetrachoric correlations

AC FI=missing_data_matrix.acc ! asymptotic covariance matrix

MO NX=10 NK=2 TD=DI, FR PH=FU, FI

LK

Main_factor Missing_data_factor

VA 1 LX 1 1

. . . . . . . . . . .

VA 1 LX 10 1

VA 1 LX 6 2

. . . . . . . . . . .

VA 1 LX 10 2

FR PH 1 1

FR PH 2 2

OU ML SC IT=1000 AD=OFF ND=3

#### The Results for Fixed Factor Loadings

**Table A1:** Mean Fit Results for One-Factor and Two-Factor Models With Fixed Factor Loadings

Model	Missing per column/overall	χ^2^	*df*	RMSEA	SRMR	NNFI	CFI	AIC
One factor	0%/0%	56.2	44	0.024	0.086	0.975	0.975	78.2
One factor	50%/25%	386.1	44	0.125	0.295	0.875	0.877	408.1
Two factors	50%/25%	73.7	43	0.038	0.090	0.988	0.989	97.7

*Note.*χ^2^ = chi-square; *df* = degree of freedom; RMSEA = root mean square error of approximation; SRMR = standardized root mean square residual; NNFI = nonnormed fit index; CFI = comparative fit index; AIC = Akaike information criterion.

#### LISREL Code for Free Factor Loadings

The code is the same as for free factor loadings (see 2.) with one exception: the code regarding factor loadings must be changed:

. . . replace . . .

VA 1 LX 1 1

. . . . . . . . . . .

VA 1 LX 10 1

VA 1 LX 6 2

. . . . . . . . . . .

VA 1 LX 10 2

by

FR LX 1 1

. . . . . . . . . . .

FR LX 10 1

FR LX 6 2

. . . . . . . . . . .

FR LX 10 2

#### The Results for Free Factor Loadings

**Table A2:** Mean Fit Results for One-Factor and Two-Factor Models With Free Factor Loadings

Model	Missing per column/overall	χ^2^	*df*	RMSEA	SRMR	NNFI	CFI	AIC
One factor	0%/0%	50.0	35	0.029	0.077	0.961	0.970	90.0
One factor	50%/25%	69.6	35	0.046	0.094	0.984	0.988	109.6
Two factors	50%/25%	59.3	31	0.043	0.101	0.985	0.990	107.3

*Note*. χ^2^ = chi-square; *df* = degree of freedom; RMSEA = root mean square error of approximation; SRMR = standardized root mean square residual; NNFI = nonnormed fit index; CFI = comparative fit index; AIC = Akaike information criterion.
